# Evaluation of pre-analytical conditions and comparison of the performance of several digital PCR assays for the detection of major *EGFR* mutations in circulating DNA from non-small cell lung cancers: the CIRCAN_0 study

**DOI:** 10.18632/oncotarget.21256

**Published:** 2017-09-21

**Authors:** Jessica Garcia, Eric Dusserre, Valérie Cheynet, Pierre Paul Bringuier, Karen Brengle-Pesce, Anne-Sophie Wozny, Claire Rodriguez-Lafrasse, Gilles Freyer, Marie Brevet, Léa Payen, Sébastien Couraud

**Affiliations:** ^1^ Laboratoire de Biochimie et Biologie Moléculaire, Centre Hospitalier Lyon Sud, Hospices Civils de Lyon, 69310, Pierre Bénite, France; ^2^ Centre de Recherche en Cancérologie de Lyon, INSERM 1052, CNRS 5286, Université Claude Bernard Lyon 1, Lyon, 69003, France; ^3^ Laboratoire Commun de Recherche Hospices Civils de Lyon – BioMérieux, Centre Hospitalier Lyon Sud, 69310, Pierre Bénite, France; ^4^ Institut de Cancérologie des Hospices Civils de Lyon, CIRculating CANcer Program (CIRCAN), 69002 Lyon, France; ^5^ Medical Diagnostic Discovery Department, BioMérieux, 69290 Craponne, France; ^6^ Service d'Anatomie et de Cytologie Pathologiques, Groupement Hospitalier Est, Hospices Civils de Lyon, 69500, Bron, France; ^7^ Faculté de Pharmacie de Lyon (IPSB), Université de Lyon1, Lyon, 69008, France; ^8^ Service d'Oncologie Médicale, Centre Hospitalier Lyon Sud, Hospices Civils de Lyon, Lyon, 69003, France; ^9^ EMR 3738 Ciblage Thérapeutique en Oncologie, Faculté de Médecine Lyon Sud, Université Lyon 1, 69600, Oullins, France; ^10^ Service de Pneumologie Aigue Spécialisée et Cancérologie Thoracique, Centre Hospitalier Lyon Sud, Hospices Civils de Lyon, Lyon, 69003, France

**Keywords:** lung cancer, EGFR mutation, circulating-free DNA, liquid biopsy, digital PCR

## Abstract

Non invasive somatic detection assays are suitable for repetitive tumor characterization or for detecting the appearance of somatic resistance during lung cancer. Molecular diagnosis based on circulating free DNA (cfDNA) offers the opportunity to track the genomic evolution of the tumor, and was chosen to assess the molecular profile of several *EGFR* alterations, including deletions in exon 19 (delEX19), the L858R substitution on exon 21 and the EGFR resistance mutation T790M on exon 20.

Our study aimed at determining optimal pre-analytical conditions and *EGFR* mutation detection assays for analyzing cfDNA using the picoliter-droplet digital polymerase chain reaction (ddPCR) assay. Within the framework of the CIRCAN project set-up at the Lyon University Hospital, plasma samples were collected to establish a pre-analytical and analytical workflow of cfDNA analysis. We evaluated all of the steps from blood sampling to mutation detection output, including shipping conditions (4H *versus* 24H in EDTA tubes), the reproducibility of cfDNA extraction, the specificity/sensitivity of ddPCR (using external controls), and the comparison of different PCR assays for the detection of the three most important *EGFR* hotspots, which highlighted the increased sensitivity of our in-house primers/probes. Hence, we have described a new protocol facilitating the molecular detection of somatic mutations in cancer patients from liquid biopsies, improving their diagnosis and introducing a less traumatic monitoring system during tumor progression.

## INTRODUCTION

In the last two decades, the discovery of somatic oncogenic drivers, such as mutations in the epidermal growth factor receptor (EGFR), has revolutionized the treatment of advanced non-small cell lung cancer (NSCLC) [1]. Some of these sensitive mutations, including L858R and exon 19 deletions (delEX19), are targetable using tyrosine kinase inhibitors (TKIs). Several EGFR-targeting TKIs are currently indicated in the front-line management of advanced NSCLC exhibiting an actionable mutation [2]. Unfortunately, most patients progress after a median progression-free survival time of around 9-12 months [2]. This tumoral progression may be due to the acquisition and/or activation of several emerging oncogenic drivers in tumor cells [3], including the acquisition of resistance EGFR mutations, such as the T790M substitution on exon 20. Interestingly, this mutation can be successfully targeted by 3^rd^ generation EGFR TKIs, such as osimertinib (Astra-Zeneca, United Kingdom), the administration of which is currently approved in the context of advanced NSCLC [4].

The search for TKI-sensitive and TKI-resistant somatic EGFR alterations remains a major challenge for the optimal clinical management of advanced NSCLC. At the turn of the 20^th^ century, a standard was gradually established to detect these mutations in tissue biopsies using several different highly sensitive methodologies (qPCR, direct sequencing, next-generation sequencing and droplet digital PCR) [5] [6]. However, it is well-recognized that advanced lung cancer produces small tissue biopsies, when obtained through invasive procedures, such as bronchoscopy or CT-scan trans-thoracic needle core biopsy rather than surgically removed. Diagnosis is thus frequently carried out using cytological samples only (endobronchial brush/aspiration, endobronchial ultra-sound, pleural effusion), since precious tissue biopsies are sometimes too small or of insufficient quality to perform both molecular and histological profiles for the comprehensive diagnosis of the cancer (histological examination, immunohistochemistry, mutation analysis, and fluorescence *in situ* hybridization (FISH) for gene rearrangement analysis) [7]. Furthermore, most practice guidelines currently recommend the reevaluation of EGFR mutated lung cancers after progression for patients receiving 1^st^/2^nd^ generation TKI therapies, to understand resistance mechanisms and optimize the strategy in subsequent lines of treatment [2]. Hence, the advent of “liquid biopsy”, based on the detection of circulating tumor DNA in the bloodstream, has long been awaited in the field of thoracic oncology. It is now well-established that a substantial proportion of tumor DNA circulates following primary tumor cell necrosis and/or apoptosis and death of circulating tumor cells [8] [9]. Following major progresses in the development of sensitive mutation detection tools, it is now possible to detect some somatic alterations from minute quantities of cfDNA (less than 0.1%) as well as in small DNA fragments [10], thus enabling the detection of alterations in small plasma samples [11] [12]. Several techniques are currently available to detect plasma DNA, including highly sensitive PCR assays and next-generation sequencing (NGS). In the case of PCR assays, cfDNA can be explored using PCR non-digital platforms (Biocartis technology (Mechelen, Belgique, EU), AmoyDx (Amoy Diagnostics Co., Xiamen, China), cobas®EGFR Mutation Test (Roche Diagnostics, Bâle, Switzerland), and the therascreen TM®EGFR (Qiagen, Valencia, CA, USA)); as well as two digital platforms, the droplet digital PCR (ddPCR, Bio-Rad, Hercules, CA, USA) and the BEAMing® digital PCR (Sysmex Inostics, Hamburg, Germany, EU). Of note the limited sensitivity of all methods used for the detection of EGFR mutations in plasma may lead to false-negative results (especially in case of low cfDNA input).

Currently, it is well-known that solid tumors exhibit spatial and temporal heterogeneity in their molecular profile, which can be further modified during disease progression and in response to treatment. The sampling of small tissue biopsies of a unique site may therefore result in a failure to detect the biomarker either at diagnosis or during progression, due to such intratumor heterogeneity. Liquid biopsies reflect the molecular profile of the whole tumor at diagnosis, as well as its evolution during disease progression. Nevertheless, the quantity of released circulating tumor DNA (ctDNA) is highly variable and the mutated fraction represents only a small proportion of circulating free DNA (cfDNA). Therefore, a highly sensitive and specific method of detection is necessary to monitor the heterogeneity and detect low amounts of mutated DNA.

Hence, while the first decade of the 20^th^ century was dedicated to setting-up and improving molecular cancer diagnosis techniques using biopsies, the years since have focused on developing such techniques using plasma. In this context, droplet digital PCR (ddPCR) has recently emerged as a highly sensitive and quantitative approach for detecting low prevalent sequences. This droplet-based technology revolves around the parallel amplification of up to millions of individual DNA fragments within identical compartments (i.e., droplets), and sensitivity is limited only by the number of DNA molecules that can be amplified and detected (i.e., the number of PCR-positive compartments) and the false-positive rate of the mutation detection assay. However, since these ddPCR assays are relatively recent, there are still many concerns regarding the most adequate process for cfDNA analysis, including pre-analytical steps, which are particularly crucial for cfDNA [13] [14]. The aim of this paper was to implement and optimize EGFR mutation detection assays in cfDNA for routine analysis. Thus, we assessed several pre-analytical conditions, and we compared various primer and probe designs targeting wild-type (WT) and mutated EGFR genes (including “in house” and commercial assays) using the ddPCR technology. We initially validated this technology on formalin-fixed paraffin-embedded (FFPE) biospies by comparing its results with next-generation sequencing (NGS) using patients with known mutational profiles.

## RESULTS

### Match between the somatic alterations detected in solid biopsies by NGS and by ddPCR

To evaluate the concordance between NGS and ddPCR results, we assessed the level of homology between the EGFR molecular profile of 10 selected FFPE biopsies from patients with known mutational profiles using NGS and ddPCR assays. We observed 100% match between NGS data and ddPCR results, irrespective of the detection system used (see Materials and Methods section). No false-positive and -negative cases were detected. In conclusion, the ddPCR assays detected known targeted somatic alterations (Table 1), thus confirming the validity of these assays, which were then compared for their individual performance using cfDNA from a larger cohort of patients.

Table 1Concordance between EGFR mutations detected in biopsies by next-generation sequencing (NGS) and by droplet digital PCR (ddPCR)

**Table:** (A) Detection of various delEX19 deletions and T790M substitutions in 7 NSCLC patients by NGS or ddPCR using Seki's assay or our in-house assay and the corresponding detection systems (described in Figure 3)

		delEX19			
	Mutation (NGS)	Seki's method	Concordance	LT's method	Concordance
**Patient #1**	delEX19 (∆746-750)	+	100%	+	100%
**Patient #2**	delEX19 (∆746-750)	+	100%	+	100%
**Patient #3**	delEX19 (∆747-752)	+	100%	+	100%
**Patient #4**	delEX19 (∆747-751)/T790M (c.2369 c>t)	+	100%	+	100%
**Patient #5**	delEX19 (∆746-750)	+	100%	+	100%
**Patient #6**	delEX19 (∆746-750)	+	100%	+	100%
**Patient #7**	delEX19 (∆747-751)	+	100%	+	100%

**Table:** (B) Detection of various L858R and T790M mutations in 7 NSCLC patients by NGS or by ddPCR using Seki's assay exclusively

		T790M		L858R	
	Mutation (NGS)	Seki's method	Concordance	Seki's method	Concordance
**Patient #8**	L858R (c.2573c>t)	-	100%	+	100%
**Patient #9**	L858R (c.2573 c>t)	-	100%	+	100%
**Patient #10**	L858R(c.2573 c>t)/T790M (c.2369 c>t)	+	100%	+	100%
**Patient #4**	delEX19 (∆747-751)/T790M (c.2369 c>t)	+	100%	-	100%

### Reproducibility of cfDNA extraction and quantification from samples stored in EDTA

**Figure 1:**
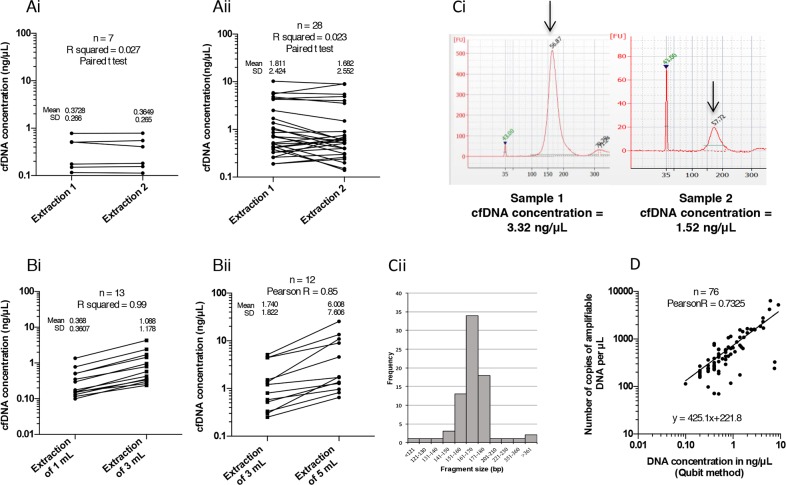
Optimization of circulating free DNA (cfDNA) extraction and quantification of cfDNA in the samples **(A)** Reproducibility of cfDNA extraction using the QIAamp Circulating Acid Kit (Qiagen, Cat No 55114, Valencia, CA, USA) on two independent cfDNA samples extracted from 1 mL **(Ai)** and 3 mL **(Aii)** of plasma from NSCLC patients. After extraction, cfDNA was quantified by Qubit dsDNA HS Assay Kit (Life Technologies, Q32854, Carlsbad, CA, USA) according to the manufacturer's instructions. **(B)** Correlation between the initial volume of plasma 1 mL *versus* 3 mL **(Bi)** or 3 mL *versus* 5 mL **(Bii)** and the quantity of cfDNA extracted (in ng/μL). **(Ci)** Fragment size visualization of cfDNA (in bp) from a concentrated (left) and a less concentrated (right) sample obtained using the Bioanalyzer (Agilent Technologies, Santa Clara, CA, USA) **(Cii)**, and average size distribution (10 bp increments) of cfDNA fragments in 77 plasma samples. **(D)** Correlation between cfDNA concentration measured using the Qubit method and the number of amplifiable copies in the corresponding plasma samples determined using the Quantifiler Kit.

The reproducibility of the QIAamp Circulating Nucleic Acid Kit was first estimated by verifying the concentration of cfDNA extracted using the Qubit Fluorometric Quantification Kit, which was previously validated using a commercial DNA solution at 0.5 ng/μL, according to the supplier's indications. We performed two independent 1 mL and 3 mL extractions from the same plasma sample. The concentration of cfDNA (ng/μL) obtained was not significantly different in the two independent extractions, either from 1 mL (n = 7; P = 0.72) or from 3 mL (n = 28; P = 0.43) of plasma (Figure 1Ai and 1Aii), thus confirming the reproducibility of the extraction kit.

Next, we assessed the efficacy of the extraction kit according to the initial plasma volume, since very low amounts of cfDNA were extracted from 1 mL plasma (ranging 0.09-4.32 ng/μL). As expected, the mean concentration increased proportionally in 3 mL compared to 1 mL samples, and in 5 mL compared to 3 mL samples, by 2.9 fold (Pearson coefficient R^2^ = 0.99; P < 10^−4^) and 3.44 fold (R^2^ = 0.85; P < 10^−4^), respectively (Figure 1Bi and 1Bii). In the latter case, this increase was unexpectedly high, reflecting two outlying values.

Since the integrity (quality) of the cfDNA conditions its amplification in ddPCR, we then analyzed the fragmentation profile of cfDNA using the BioAnalyzer technology (Figure 1Ci). The average size of cfDNA fragments was 168 bp (±SD 9.6, n. = 70) (Figure 1Cii). In addition, 85% of the samples contained cfDNA fragments ranging between 150 bp and 180 bp (Figure 1Cii). This implies that for amplicons larger than 180 bp, the performance of the ddPCR assay would be decreased. Consequently, all samples could be directly analyzed by ddPCR without DNA pre-fragmentation, which is highly recommended by Bio-Rad for the amplification of genomic DNA.

We also verified whether cfDNA fragments were amplifiable by qPCR using the Quantifiler Human DNA Quantification Kit. This qPCR methodology provides a correlation between the quantity of cfDNA expressed in ng/μL and a number of gene copies of hTERT/μL. The concentration of cfDNA measured by Qubit and the quantity of amplifiable DNA were highly correlated (n = 76; Pearson test R^2^ = 0.73; P < 10^−4^) (Figure 1D). Only a few samples were poorly amplified (Figure 1D), unlike FFPE DNA extractions that are frequently of insufficient quality to produce adequate amplifications.

Based on these results and for the purpose of routine applications, we defined a minimum plasma volume for cfDNA extraction of 3mL. Indeed, this volume was chosen for the following reasons: (i) use of 4 × 5 mL EDTA tubes, provides analysts with approximately 6 mL of plasma per patient; (ii) we have shown above that 1 mL plasma samples were inadequate for our analyses that requires at least 3mL; (iii) for routine diagnosis use, we always divide our samples in two in order to have a backup in the event of mishandling or contamination. This back-up sampling also enabled us to duplicate all our tests so as to respect insurance quality guidelines in terms of standardization, accuracy and reproducibility of our assays; (iv) and we cannot use 5 mL unless we collect 8 EDTA tubes per patient which constitutes a heavy burden for the patient.

### Assessment of blood sampling and shipping conditions

**Figure 2:**
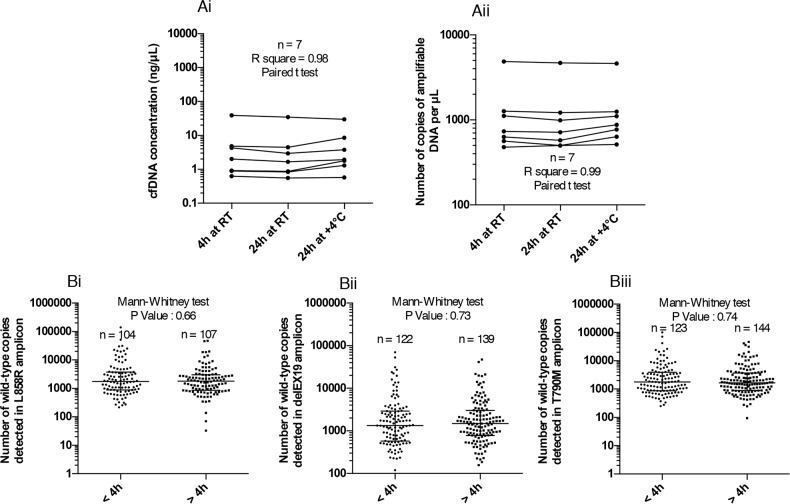
Impact of pre-analytical blood storage conditions in EDTA tubes on circulating free DNA (cfDNA) integrity **(A)** Evaluation of the effect of blood storage time (4 hours or 24 hours) and temperature (room temperature RT or 4°C) in EDTA tubes prior to plasma collection, on the concentration (ng/μL) of cfDNA extracted using the Qubit Quantification Kit **(Ai)**, and the number of amplifiable DNA copies using the Quantifiler technique **(Aii)**. Blood samples from the same patient (n = 7) were processed according to the three storage conditions. **(B)** Exploration of the number of wild-type (WT) copies of 3 independent regions of *EGFR* gene with the different ddPCR systems detailed in Figure 3 for WT L858R **(Bi)**, WT delEX19 **(Bii)** and WT T790M **(Biii)** when samples are processed within 4 hours and within 24 hours after blood sampling (data not paired).

To determine the impact of EDTA sampling and shipping conditions on the stability of cfDNA, we tested three different pre-analytical blood storage conditions in K_2_EDTA tubes before cfDNA extraction: (i) 4 hours at room temperature, (ii) 24 hours at room temperature (iii) and 24 hours at 4°C. Our aim was to verify whether EDTA storage/shipping could be used up to 24 hours to facilitate the transfer between hospitals and analytical platforms. CfDNA extraction and quantification were thus carried out independently. CfDNA concentrations were determined by Qubit quantification (Figure 2Ai) and amplifiable DNA was measured by qPCR as described above (Figure 2Aii). The cfDNA concentration (n = 7, One-way ANOVA, R^2^ = 0.9812, P < 0.0001) and amplifiable cfDNA fraction (n = 7, One-way ANOVA, R^2^ = 0.9978, P < 0.0001) were not statically different, irrespective of blood storage conditions (Figure 2Ai and 2Aii). To support these findings, we compared the number of WT copies in our three regions of interest in 104 to 144 unpaired patients' blood samples processed either within 4 hours (samples from our hospital), or between 4 hours and 24 hours (collected in others hospitals). No statistical change in the number of cfDNA WT copies was observed between samples processed within 4 hours and within 24 hours, irrespective of the *EGFR* regions studied and the shipping conditions (Figure 2B). To support these observations, we compared the number of positive cases (whatever the alteration) of EGFR mutations between samples processed before 4 hours (n=163) and after 4 hours (< 24 hours; n=190). We found a very similar rate of 25.7% and 25.3% respectively.

### Comparison of the accuracy of the three systems for the detection of somatic EGFR alterations

**Figure 3:**
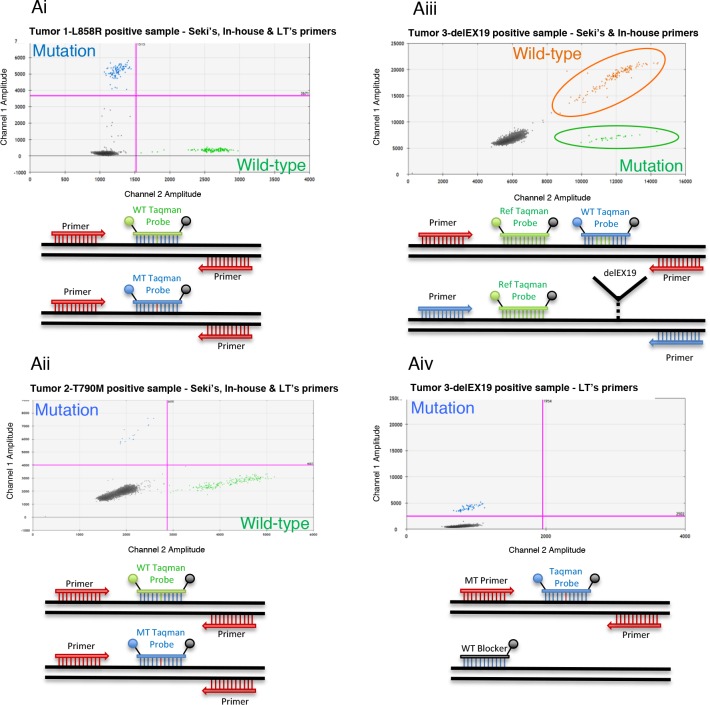
Overview of three Droplet Digital PCR (ddPCR) detection systems **(Ai-Aiv)** Detection of 3 *EGFR* somatic alterations: L858R and T790M substitutions and delEX19 deletions. Top, 2D flow cytometry plots; bottom, schematic diagrams showing the principles of the corresponding ddPCR detection systems. **(Ai-Aii)** Dual probe system used to detect L858R **(Ai)** and T790M mutations **(Aii)** from liquid biopsies of NSCLC patients. This system can be used for the three detection assays described in Table 2, namely Seki's, Life Technologies' or our in-house assay. It is based on the utilization of reverse and forward primers targeting the hotspot and 2 taqman probes (WT and MT) labeled with 2 distinct fluorophores, VIC and FAM. The first anneals to wild-type (WT) copies whereas the latter binds to mutated (MT) copies. **(Aiii)** Dual labeling system used to detect delEX19 deletions with Seki's (Seki et *al*., 2016) or our in-house detection primers/probes. This system revolves around the dual labeling of WT copies (by VIC and FAM) and single labeling of MT copies (by VIC only). **(Aiv)** A single probe system used to detect delEX19 deletions with Life Technologies' assay (Hs00000228_mu), which is designed to detect only MT copies by blocking the WT sequence with a blocker.

**Figure 4:**
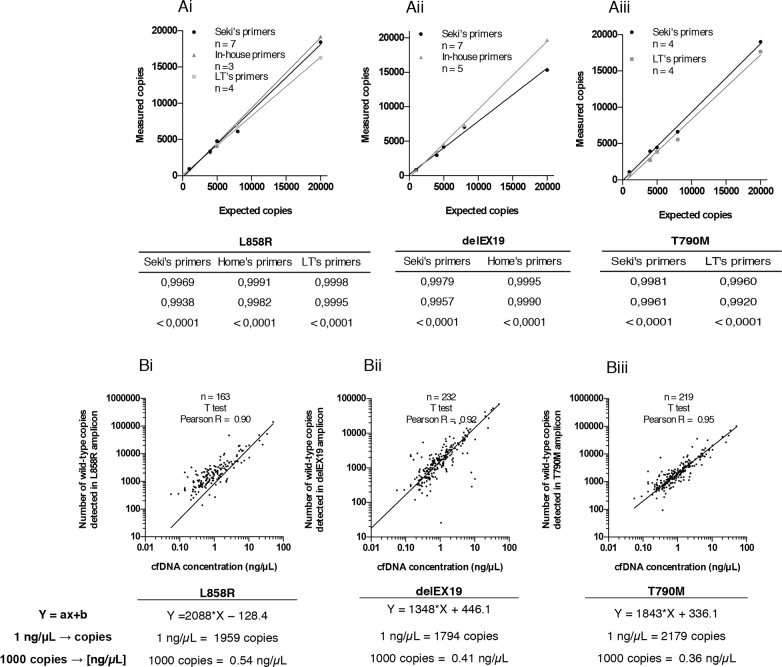
Accuracy of the three Droplet Digital PCR (ddPCR) systems used **(A)** Top, correlation between the theoretical expected number of wild-type (WT) copies and experimentally measured WT copies of commercial genomic DNA from the Quantifiler Human DNA Quantification Kit (Applied Biosystems, PN4344790F, Foster City, CA, USA) by ddPCR for the detection of **(Ai)** L858R substitutions, **(Aii)** delEX19 deletions and **(Aiii)** T790M substitutions, according to the detection assay used, namely Seki's assay, Life Technologies' (LT's) assay, or our in-house assay. Bottom, tables summarizing statistical data presented above. **(B)** Top, correlation between the number of WT copies for **(Bi)** L858R substitutions, **(Bii)** delEX19 deletions and **(Biii)** substitutions T790M and the concentration of cfDNA (in ng/μL) measured by Qubit (Life Technologies, Q32854, Carlsbad, CA, USA) in cfDNA samples. Bottom, equation used to estimate the concentration of cfDNA required to detect a threshold level of 1,000 mutated copies, for each plot presented above.

We then evaluated the number of copies of the three *EGFR* regions mentioned above obtained using two (T790M) or three (L858R and delEX19) independent mutation detection assays, namely Seki's [15] and an in-house assay for L858R and delEX19, or the latter two and Life Tech's assay for all three mutations (see Materials and Methods section, Table 1 and Figure 3Ai-3Aiv). To test the accuracy of these systems, we used a commercially available genomic DNA (60,600 copies/μL provided with the Quantifiler Kit) at different concentrations (20,000 copies/μL to 1,000 copies/μL). We observed a strong correlation between the expected genomic DNA input and the experimental number of WT copies quantified by all three systems (Figure 4Ai-4Aiii). A strong correlation between Qubit quantification and the number of WT copies for the three regions was also obtained (Figure 4B).

Next, we calculated the cfDNA concentration expressed in ng/mL representing 1,000 WT copies in 3 mL of patient plasma for each EGFR region. The average cfDNA concentration required for the detection of the mutations was 0.54 ng/μL for the L858R substitution (Figure 4Bi), 0.41 ng/μL for the delEX19 (Figure 4Bii) and 0.36 ng/μL for the T790M substitution (Figure 4Biii).

### Specificity of the different detection systems

**Figure 5:**
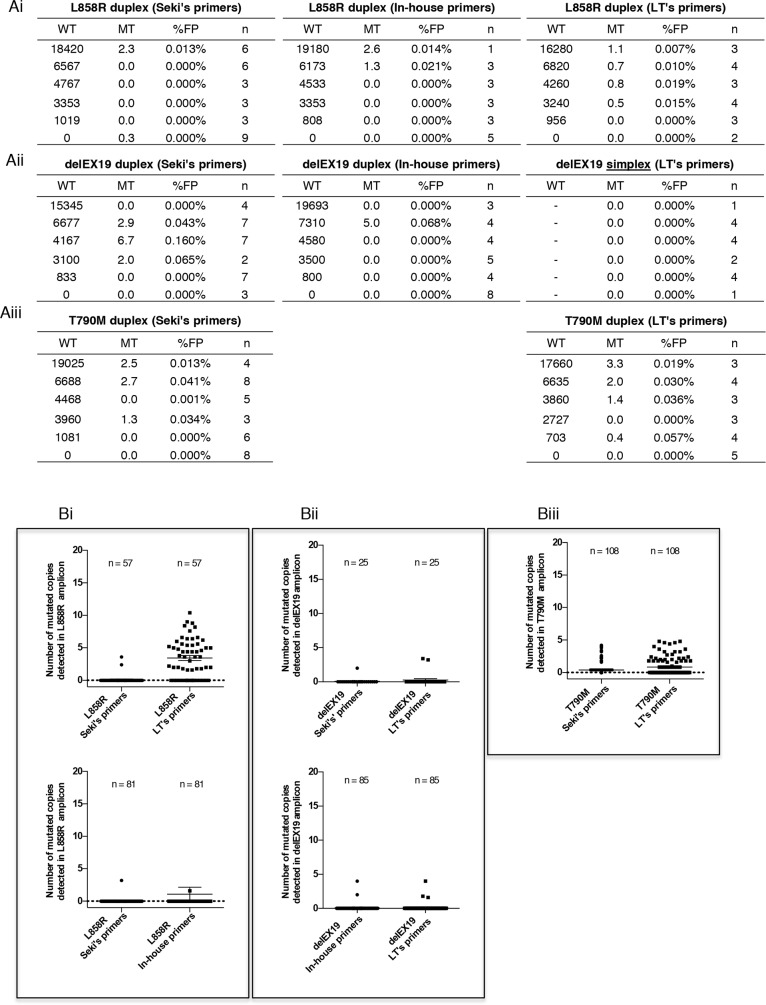
Specificity of the three Droplet Digital PCR (ddPCR) systems used **(A)** Determination of false-positive cases (mutated MT) detected using the three ddPCR systems described in Figure 3 and a commercial genomic wild-type DNA control provided in the Quantifiler Human DNA Kit (Applied Biosystems, PN4344790F, Foster City, CA, USA). The commercial WT DNA was diluted and tested for L858R substitutions **(Ai)**, various delEX19 deletions **(Aii)** and T790M substitutions **(Aiii)** using three detection assays: Seki's assay, an in house's system and LT's system (see Table 2); n indicates the number of independent experiments carried out for each conditions. WT and MT colums indicate the mean of absolute detected copies. The numbers and rates of false-positives (% FP) cases are reported. **(B)** Background of false-positive copies (%MT) for all of the ddPCR mutation systems used to detect L858R substitutions **(Bi)**, various delEX19 deletions **(Bii)** and T790M substitutions **(Biii)** from cfDNA of NSCLC patients with a negative or unknown biopsy status at diagnosis and with negative results in ddPCR. The absolute copy number was based on the maximum number of MT copies observed in tables Ai-Aiii (5 MT copies) over the minimum WT detection threshold (500 WT copies).

The specificity of the three detection systems, was determined by verifying the number of false-positive cases detected using the ddPCR technology and commercially available DNA Quantifiler. We found that the maximum number of falsely detected MT copies was below to 5 copies - irrespective of the quantity of WT DNA input - in most of assays (Figures 5Ai to 5Aiii) except one outlying value for delEX19 in-house assay and one outlaying value in Seki's delEX19 assay. Interestingly, 5 MT copies correspond to a mutation fraction of 1% in case of the detection of 500 WT copies which constitutes our minimum detection threshold in 3 mL of plasma. Thus, we found that the absolute copy number falsely MT was similar whatever the amount of genomic WT DNA.

To consolidate these data, since cfDNA is fragmented and its quality differs from the commercial samples, we measured the number of false-positive copies from patient cfDNA with known WT molecular profiles in biopsies at diagnosis (Figure 5Bi-5Biii). Samples were paired for each assays. All systems found < 5 falsely absolute mutated copies, underlining their high level of specificity, except for LT's L858R system (Figure 5Bi). Since the manufacturer does not provide details for this kit, we can only speculate that either the primers or probes for this kit had difficulties annealing to fragmented patient cfDNA.

### Sensitivity of assays for the detection of somatic alterations

**Figure 6:**
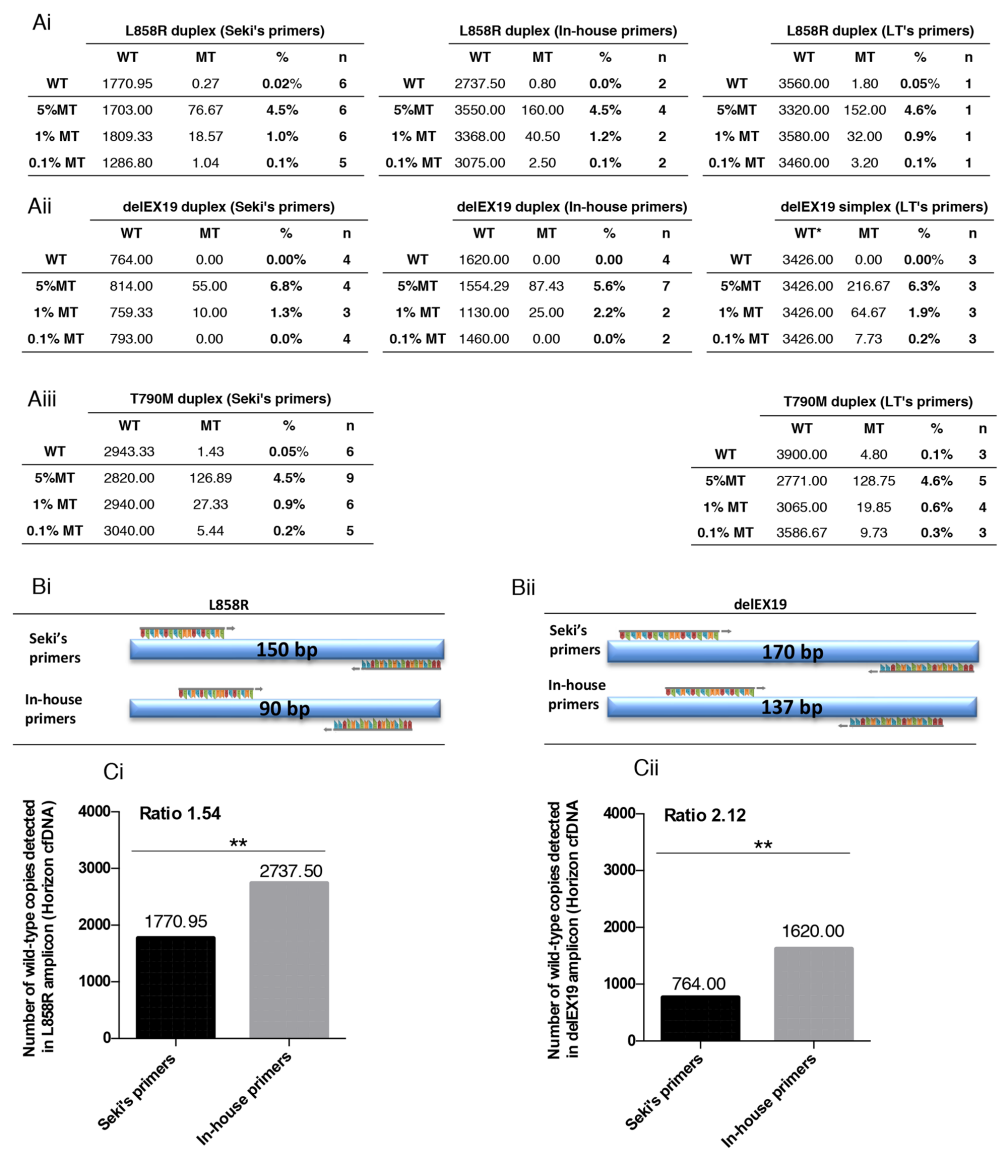
Sensitivity of the three Droplet Digital PCR (ddPCR) systems used Correlation between measured mutated (MT) and wild-type (WT) copies with the theoretical percentage of mutated copies of four reference standards DNA (Horizon Diagnostics) for three somatic *EGFR* alterations L858R and T790M substitutions, and various delEX19 deletions detected using the ddPCR systems described in Figure 3 and Table 2. The commercial standard DNA was tested for **(Ai)** L858R mutations with Seki's, Life Technologies' (LT's) and our in-house primers/probes. **(Aii)** delEX19 deletions were detected using the same three systems, while **(Aiii)** T790M mutations were detected using Seki's and LT's primers/probes. **(B)** Representation of the size of the amplicons generated during ddPCR with Seki's primer and our in-house primers for the L858R **(Bi)** and delEX19 **(Bii)** gene regions. n indicates the number of independent experiments carried out for each conditions. WT and MT colums indicate the mean of absolute detected copies. **(C)** Histogram presenting the number of WT copies detected using Seki's and LT's primers/probes for L858R mutations **(Ci)** and delEX19 deletions **(Cii)**. The ratio represents the difference in the number of MT copies detected between our in-house primers and Seki's primers.

To evaluate the sensitivity of the different assays, we used Horizon's cfDNA standards, since they are well-characterized and routinely used as reference materials to assess the performance of cfDNA assays. Horizon's Multiplex I cfDNA Reference Standard Set covers multiple engineered single nucleotide variants with eight mutations at predefined levels of 5%, 1%, and 0.1% allelic frequencies. Of note, the mean size of Horizon's cfDNA standard is 160 bp according to the manufacturer's indications. We compared the sensitivity of these assays for the detection of L858R, delEX19 and T790M mutations (Figure 6Ai-6Aiii), and mainly estimated the detection threshold at 1% (according to cfDNA input), except for LT's delEX19 and T790M systems (Figure 6Aii, right and 6Aiii, right) and Seki's T790M system (Figure 6Aiii, left) that were even more sensitive for these mutations at 0.1%.

Owing to the fact that cfDNA is highly fragmented and its mean fragment size is 160 bp, we speculated that the length of amplicons may strongly impact the performance of the detection assays. Since the amplicons of Seki's T790M detection assay are under 100 bp, we did not design any in-house primers for this mutation. In contrast, the amplicon lengths are 150 bp and 90 bp for Seki's assay and our in-house assay, respectively, for the detection of the L858R mutation (Figure 6Bi), resulting in the latter case in a significant increase in the number of WT copies detected using Horizon's cfDNA standard (Figure 6Ai-6Aii). Similarly, our in-house amplicon for delEX19 are shorter than Seki's by 33 bp (Figure 6Bii), resulting once again in an almost 2-fold increase in the number of WT copies detected. Thus, the number of amplifiable WT copies is inversely correlated with the length of the amplicons used in the assays, and Seki's detection assay amplified fewer WT copies compared to our own design in Horizon's cfDNA standard (Figure 6C).

**Figure 7:**
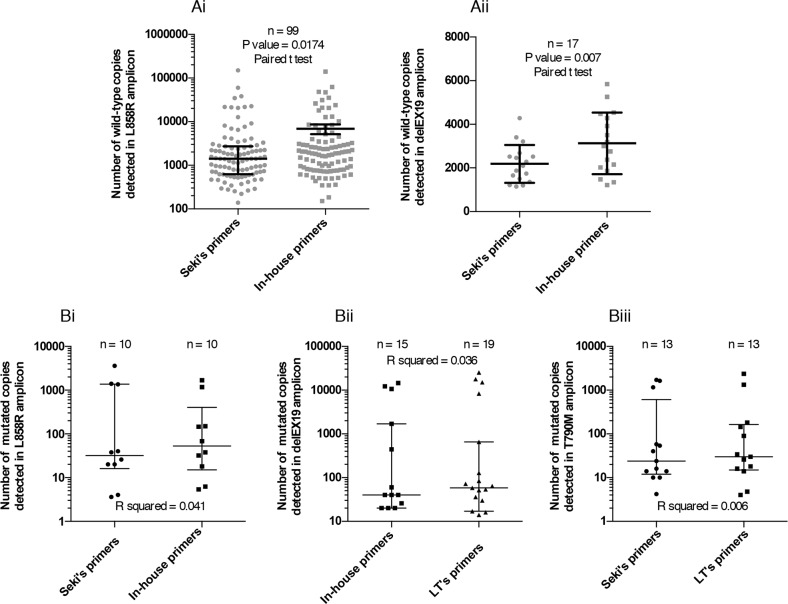
Range of the number of mutated copies detected by Digital Droplet PCR (ddPCR) **(A)** Representation of the range of WT copies using Seki's primers and our in-house primers for the detection of L858R **(Ai)** and delEX19 **(Aii)** among patients with or without a positive mutation status at diagnosis. **(B)** Comparison between pairs of detections systems used to evaluate the number of mutated copies among patients harboring EGFR alterations for: L858R **(Bi)**, various delEX19 **(Bii)** and T790M **(Biii)**. μ

Consistently, the number of copies of corresponding WT forms for L858R (Figure 7Ai) and delEX19 (Figure 7Aii) in patient cfDNA samples displayed a similar correlation.

We eventually evaluated the number of mutated copies of three regions of *EGFR* in patient cfDNA with known mutations at diagnosis, using the two best assays for each region (i.e., excluding the LT's assay for L858R (see Figure 5Bi) and Seki's assay for delEX19 (see Figure 6Cii) due to their lower performances). We found no difference between each assay pairs in the three regions (Figure 7B).

## DISCUSSION

Non invasive methods for the detection of somatic mutations are currently useful and relevant at diagnosis and during tumor progression in lung cancer. In this context, liquid biopsies enable biologists, pathologists and clinicians to determine the genetic landscape of the whole tumor (primary and metastatic), and provide them with the opportunity to systematically track genomic evolution [8]. Indeed, molecular somatic changes in cfDNA have frequently been associated with tumor burden [9]. This molecular profiling is mandatory to choose the best treatment for each patient [5]. The aim of our current work was to implement the detection of somatic alterations from cfDNA in routine diagnosis, using highly sensitive and specific detection assays, such as digital PCR.

To consolidate the pre-analytical workflow of cfDNA analysis, we studied the stability of sampling in EDTA tubes and the effect of total blood sampling time on cfDNA integrity. Indeed, most of the suppliers' instructions and guidelines currently recommend either the use of tubes with nucleated-cell stabilizers (such as the Cell-free DNA BCT tube (Streck Inc., La Vista, NE, USA) or the PAXgene Blood ccfDNA tube (Qiagen, Valencia, CA, USA), or the processing of the samples within 4 hours in EDTA tubes [13] [14] [16] [17]. However, tubes containing membrane stabilizers are expensive (up to 50 times the price of EDTA tubes) and their use for routine diagnosis is not validated in some countries, such as in the US. In addition, the use of EDTA tubes would greatly facilitate the integration of cfDNA analysis in a classical pre-analytical routine workflow, since these tubes are widespread and currently used on a systematic basis. By contrast, the need for specific pre-analytical conditions would rely either on the blood sampling of patients in specific (centralized) sampling laboratories, thus requiring patients to travel sometimes frequently during their illness, or on the training of local biology/medical laboratories in blood sample processing (preliminary steps) prior to sending frozen plasma to the molecular diagnosis laboratory. Both cases present limitations and are major barriers for the routine implementation of diagnosis from cfDNA. Furthermore, very few studies have examined the effect of sampling and shipping conditions on the final quality of the cfDNA extracted, particularly with regards to processing within 24 hours. Several authors comparing the sampling tubes and time of analysis, reported no significant difference in mean cfDNA concentration after sampling in EDTA and BCT [18] [19] and analyzing within 6 hours. However, in these studies EDTA tubes exhibited poor performances after 72 hours and 14 days compared to tubes containing nucleated-cell stabilizers [18] [19]. In a paper by Kang *et al*. [20], performances of EDTA, PAXgene Blood ccfDNA, and BCT tubes were determined in 10 patients for the detection of cfDNA mutations. In agreement with our findings, performances were similar in the three tubes at 2 hours and 6 hours, but EDTA tubes exhibited poor performances at 48 hours (the 24 hour time period was not tested). Taken together with our own results at 24 hours, these studies substantiate the fact that hemolysis (by measuring the absorption of free hemoglobin in plasma at 414 nm) of whole blood stored in EDTA tubes increases after 24 hours, highlighting the need to use tubes with nucleated-cell stabilizers for processing after this time period [13] [18] [20] [21].

Interestingly, Norton *et al.* also studied the effect of shaking tubes (150 rpm on an orbital shaker, reproducing the pneumatic shipping usually used for in-house transportation within a hospital) and of real shipping conditions (analysis performed within 4 days, the 24 hour time point was not tested) [18], highlighting the well-known increase in the release of cfDNA and the better performance of BCT tubes. Here, we provide data supporting the use of EDTA tubes for up to 24 hours following blood sampling for the routine search of somatic alterations on cfDNA, since no release of genomic DNA was observed at 24 hours following shipping by car at room temperature.

To obtain sufficient sensitivity to detect somatic alterations in plasma, we used the Digital Droplet PCR (ddPCR) technology, which is widely used in oncology, including in the search for EGFR resistance mutations [22] [23], the monitoring of the kinetics of sensitivity/resistance mutations under treatment [24] [25] [26], or the study of copy-number variation (CNV) [27] [28]. Furthermore, since the performance of the detection system is widely conditioned by cfDNA input, this sensitivity highlights the importance of the cfDNA extraction step. Based on this concept and on a previous report by Devonshire *et al.* [29], who compared three commercial extraction kits, and clearly showed the higher efficacy of the QIAamp circulating nucleic acid extraction kit (Qiagen), we conducted all extraction steps using this kit. This kit enabled us to isolate cfDNA from plasma with a higher output concentration and greatly limited the contamination by large fragments (over 200 bp) of genomic DNA. In-house (Figure 1), we validated linearity between the concentration of cfDNA extracted and plasma volume. We also validated that the extraction kit excludes cfDNA fragments exceeding 200 bp (95% fragments < 200bp in 77 patients) (Figure 1Cii) and results in a mean fragment size of 163bp +/− 26 bp Mean ctDNA fragments are known to range between 145-160 bp [9] [10]; and recent findings indicate that the mean fragment size is 20 bp shorter for ctDNA than cfDNA from healthy cells [32]. In addition, we found that total amount of cfDNA, and total amount of amplifiable DNA did not differ regarding sample processing (4 hours vs. 4- 24 hours, cf. Figure 1). Taken together, these data clearly indicate that there was little genomic release of cfDNA regardless of processing conditions.

Next, we evaluated the accuracy of the ddPCR system by comparing the number of measured copies versus the expected number of copies in three EGFR regions using commercial genomic DNA. As described by Watanabe *et al.* [30], a linear correlation was found between the expected and experimental values in a wide dynamic range (0-20,000 copies) for the three wild-type regions of EGFR, using several mutation detection primers/probes (reported by Seki *et al.* [15], supplied by Life Technologies, or designed in-house) and the ddPCR method. Moreover, the specificity of our assays using commercial WT DNA was ≤ 4 copies except for Seki's delEX19 assays. These findings led us to set-up a threshold in absolute copy number (above 4 MT copies (≥ 5)) to positively define a mutated sample, independently of the amount of the DNA input. This threshold corresponds to a 1% mutated fraction in case of a DNA input of 500 WT copies, but 0.1% for 5000 copies or 0.01% of 50000 copies. However, in patient cfDNA samples (WT at biopsy) results were slightly different (Figures 5B). Sekis's assays performed better than LT's L858R assays. Tumor heterogeneity (WT in FFPE biopsy but true-MT in blood) cannot explain these differences since our samples were paired. These findings emphasise the fact that in-house testing and comparison of detection assays is crucial, even in the case of commercial assays which may not always perform as anticipated. Commercial probes and primers should also be studied carefully and can be perfected in-house to increase the efficacy of the assays. For the T790M assays, we obtained few cases with bordeline values around the threshold of 4 absolute copy numbers. However, some cfDNA samples slightly exceeded our defined threshold of 4, while they clearly remained under the limit of detection in the paired corresponding assay. We cannot exclude that a T790M clone is emerging as recently underlined by Sacher *et al*. [31]. To compare the sensitivity of the mutation assays, we then used the control commercial cfDNA provided by Horizon Diagnostics. Under our experimental design and in agreement with the pre-analytical routine workflow in terms of cfDNA input, six detection systems of the eight tested (included the detection of the three EGFR mutated regions using the three sources of primers/probes listed above, except for the T790M substitution using in-house primers/probes) were able to detect mutated forms at a threshold level ranging from 0.1% (Life Technologies assays being the highly sensitive) to 1%.

Circulating tumor DNA (ctDNA) shed into the bloodstream by malignant cells or dying (apoptosis/necrosis) cells are fragmented with a mean size of 145-160 bp [9] [10]. Indeed, in agreement with the literature [32], in which an average size of 160 bp has been reported, we obtained a value of 168 bp ((±SD 9.6; n. = 70) from plasma tested in our hospital center. This corroborates a recent study revealing that the mean fragment size is 20 bp shorter for ctDNA (circulating tumor DNA) than cfDNA from healthy cells [33]. Moreover, we designed in-house primers for the detection of L858R and delEX19 mutations resulting in shorter amplicons. Consequently, we were able to decrease the size of the amplicons by 40% for the detection of L858R (90 bp) and 20% for the detection of delEX19 copies (137 bp), and to greatly increase the performance of the ddPCR in terms of number of wild-type copies obtained using the of the gold-standard kit from Horizon (Figure 6) and supported by findings on cfDNA from patient plasma (2-fold increase in the number of wild-type copies detected).

In summary, the optimal conditions identified in our study for the implementation of cfDNA analysis in routine diagnosis were (i) the sampling of patient blood in EDTA tubes and analysis within 24 hours, (ii) the extraction of cfDNA from at least 3 mL plasma using the QIAamp circulating nucleic acid kit from QIAgen to obtain a sufficient cfDNA input for the detection of 1,000 wild-type copies, (iii) the use of a highly sensitive and specific Droplet Digital PCR technology (Bio-Rad), (iv) the in-house design of primers/probes to amplify shorter amplicons and increase the sensitivity of the assay, and (v) the limitation of false-positive case by implementing for each targeted hot-spot, two distinct detection systems. Overall, our results argue in favor of the routine use of digital PCR, as pointed out within the AURA trial (NCT01802632, funded by Astra Zeneca) focusing on the detection of EGFR alterations in plasma by digital PCR (ddPCR and BEAMing), either as a complementary technique to tissue biopsies for the detection of mutations at diagnosis or as an alternative method for repeated analyses during tumor progression [25]. Furthermore, a precise quantitative detection technology may improve disease interpretation and may be associated with clinical outcome, although clinically relevant thresholds have to be defined. Hence, we believe that we have established a new protocol to facilitate the molecular detection of somatic mutations in cancer patients from liquid biopsies, thus greatly improving their early diagnosis and introducing a less traumatic monitoring system during tumor progression.

## MATERIALS AND METHODS

### Cohort description

Samples were collected within the framework of the CIRCAN_Lung (“CIRculating CANcer”) study, which is a prospective program established to setup the analysis of biomarkers in cfDNA, in order to implement the routine diagnosis of EGFR alterations. Between June 2015 and April 2016, 200 lung cancer patients were included in this study.

The main inclusion criteria were (i) that patients were histologically or cytologically diagnosed as having metastatic non-small cell lung cancer (NSCLC), and (ii) that these patients had undergone molecular testing for EGFR in tumor biopsies (as usually performed in France) (5). Patient inclusion was initially limited to patients treated in our center.

We then included patients treated in other centers of the *Auvergne-Rhône-Alpes* region. At this stage, however, patient inclusion was restricted to (i) patients having had no molecular testing of EGFR in tumor biopsies at diagnosis, and (ii) patients with known EGFR mutations, under EGFR TKI treatment and with a record of disease progression.

### Ethics approval

The CIRCAN study was considered to be an observational study by the local ethics committee of Lyon (Ref L15-74; 04/29/2015). As required, the study was declared to the local authorities, since patient health data were recorded (Ref 15-045; 05/15/2015). Furthermore, all of the patients were given detailed information about the present study and signed a written consent form.

All of the samples and medical data used in the CIRCAN study were anonymized. CfDNA extraction and ddPCR analyses were performed by investigators who did not have access to clinical data and were unaware of the therapeutic outcome of the patients.

### Sample collection

Formalin-fixed paraffin embedded (FFPE) biopsy tumor tissues (n = 10) were collected and served as controls for the detection of EGFR mutations. For plasma samples, 30 mL of total blood were collected in K_2_EDTA tubes (BD, 367525, 18 mg) and centrifuged for 10 min at 1,600 g. For each sample, the pellet containing leukocytes and red blood cells was discarded, while the supernatant was further centrifuged at 6,000 g for 10 min. The plasma was harvested in 2 mL cryotubes (NUNC) and stored until further use at −80°C.

### DNA extraction

FFPE tumor samples were microdissected (microdissector LMD2000, Leica, Germany, EU) to select areas of the sample with the highest percentage of tumor cells and the smallest amount of normal tissue. Hence, samples were constituted of at least 70% of tumor cells. Tumor DNA was then extracted from microdissected tissues using the QIAamp DNA FFPE Tissue Kit (Qiagen, Valencia, CA, USA), according to the manufacturer's instructions. These samples were then analyzed using customized ampliseq library and next-generation sequencing (NGS) (PGM, Life Technologies, Carlsbad, CA, USA).

CfDNA was extracted from 1 mL, 3 mL or 5 mL of plasma using the QIAamp Circulating Nucleic Acid Kit (Qiagen, Cat No 55114, Valencia, CA, USA), with a Qiagen vacuum manifold following the manufacturers' instructions. CfDNA was then eluted in a final volume of 60 - 110 μL elution buffer (AVE), depending of the volume of plasma used for the extraction (1 mL, 3 mL or 5 mL).

### CfDNA fragment size assessment and cfDNA quantification

To evaluate the size distribution of cfDNA fragments, samples were assessed using the Agilent 2100 BioAnalyzer (Agilent Technologies, Santa Clara, CA, USA) and the DNA HS kit (Agilent Technologies, Santa Clara, CA, USA, 5067-4626 & 5067-4627). Each sample was compared with two size-standardized internal controls (of 35 bp and 10,380 bp) and a DNA ladder (15 peaks). The profile of fragment sizes was generated using the 2100 Expert Software (Agilent Technologies, Santa Clara, CA, USA).

To determine the concentration of cfDNA, two blinded independent complementary assays were carried out. First, the quantification of double-strand DNA was assessed using a Qubit 2.0 Fluorometer and the Qubit dsDNA HS Assay Kit (Life Technologies, Q32854, Carlsbad, CA, USA) according to the manufacturer's instructions. The concentration was expressed in ng/mL and then converted to ng/μL. We also quantified amplifiable cfDNA by quantitative PCR (qPCR) using the Quantifiler Human DNA Quantification Kit (Applied Biosystems, PN4344790F, Foster City, CA, USA) according to the manufacturer's instructions, with the *hTert* gene (human telomerase reverse transcriptase). Based on the *C*_T_ value of the internal positive control (IPC), we determined both the number of copies of gene/mL of plasma or ng/μL of cfDNA of our samples.

### Description of the mutation detection assays

We compared the performance of three different ddPCR systems to detect three EGFR somatic alterations, namely the T790M (c.2369C>T) substitution in exon 20, the L858R (c.2573T>G) substitution in exon 21, and several deletions in exon 19 (delEX19): p.K745_E749del, p.K745_T751>K, p.E746_R748>E, p.E746_A750del, p.E746_A750>IP, p. E746_T751>IP, p.E746_T751>I, p.E746_T751>V, p. E746_T751>A, p.E746_T751>VA, p.E746_751T>E, p. E746_T751del, p.E746_S752>I, p.E746_S752>A, p.E746_S752>V, p.E746_S752del, p.E746_S752>D, p.E746_P753>VS, p.E746_S753del, p.E746_A755>E, p.L747_E749del, p. L747_A750del, p. 747_A750>P, p. L747_T751del, p.L747_T751>Q, p. L747_T751>P, p. L747_T751>S, p. L747_S752del, p. L747_S752>Q, p.L747_P753del, p.L747_P753>V, p.L747_P753>S, p. L747_P753>Q, and p.S752_I759del.

These systems were based on the use of different sets of primers and probes (Table 2) to amplify and fluorescently highlight the specific mutations by flow cytometry. These primers and probes were either those published by Seki *et al.* [15] (so forth designated as Seki's assay), those supplied by Life Technologies (AHRSRSV EGFR 6224, Hs00000228_mu, AHRSROS EGFR 6240, designated as Life Tech.'s or LT's assay), or designed in-house (in-house assay). The detection of the point-substitutions (L858R and T790M), either using Seki's assay or our in-house assay, relied on a dual probe system. Indeed, a fluorescent VIC probe bound to the wild-type (WT) DNA locus, while a fluorescent FAM probe bound to the mutated DNA locus (Figure 3Ai and 3Aii). Of note, our in-house primers were designed to amplify a shorter sequence (90 bp instead of 150 bp) than Seki's primers for the L858R mutation, and since the size of the amplicon was already quite short for the T790M (96 bp) using the latter assay, we refrained from designing our own in-house primers for this mutation. Life Technologies' Kits (AHRSRSV EGFR 6224 and AHRSROS EGFR 6240) resulted in a similar bi-fluorescent labeling, although the company does not disclose any information about their kits.

**Table 2:** Description of the mutation detection systems tested

*Seki's method*	L858R (c.2573 T>G)	delEX19	T790M (c.2369 C>T)
Forward	ACTTTGCCTCCTTCTGCATGG	GCACCATCTCAC AATTGCCAG	CGCCTGCTGGGCATCTG
Reverse	CTACTTGGAGGACCGTCGC	CACAGCAAAGCA GAAACTCACA	GTCTTTGTGTTCCCGG ACATAGT
WT - Probe	VIC - AGTTTGGCC**A**GCCCAA - MGB	VIC-CAGAAGGTGAG AAAGTT-MGB	VIC - ATGAGCTGC**G**T GATGAG - MGB
MT - Probe	FAM - AGTTTGGCC**C**GCCCAA - MGB	FAM-ATGTTGCT TCTCTTAATTCC-MGB	FAM - ATGAGCTGC**A**T GATGAG - MGB
Amplicon Size	**150 bp**	**170 bp**	**96 bp**
***LT's method***			
Kit Reference	Kit Reference (duplex)	Kit Reference (simplex)	Kit Reference (duplex)
AHRSRSV EGFR 6224	Hs00000228_mu	AHRSROS EGFR 6240	
Amplicon Size	ND	ND	ND
***In-house method***			
Forward	TGGTGAAAACACCGCAGCAT	ATTGCCAGTTAACGTCTTCC	
Reverse	CTCCTTCTGCATGGTATTCTTTC	CATCGAGGATTTCCTTGTTG	
WT - Probe	VIC - AGTTTGGCC**A**GCCCAA - MGB	VIC-CAGAAGG TGAGAAAGTT-MGB	
MT - Probe	FAM - AGTTTGGCC**C**GCCCAA - MGB	FAM-ATGTTG CTTCTCTTAATTCC-MGB	
Amplicon Size	**90 bp**	**137 bp**	

In the case of delEX19, two systems were used. The first system using Seki's or our in-house primers/probes relied on the dual labeling of the WT DNA locus and single labeling of the mutated DNA locus. Indeed, the VIC labeled probe bound near the deletion hot spot (binding WT or delEX19), while the FAM labeled probe bound specifically to the undeleted WT region, resulting in dual labeled FAM/VIC WT PCR droplets and VIC only labeled mutated droplets (Figure 3Aiii). These assays result in the detection of 19 delEX19 mutations, and once again our in-house primers were designed to amplify a shorter sequence (137 bp instead of 170 bp) than Seki's assay [15]. The second system based on the TMDA (Taqman Mutation Detection Assays) strategy of Life technologies (HS_00000228_mu) is composed of a probe annealing mutated forms exclusively and a blocker probe for WT forms (Figure 3Aiv), thus leading to the detection of WT mutated copies only.

### Amplification by picodroplet digital PCR (ddPCR)

Amplification of all of these EGFR somatic alterations was achieved using the highly sensitive and quantitative Droplet Digital PCR (ddPCR™, Bio-Rad/MolecularMD, Hercules, CA, USA). Briefly, BioRad's QX100 ddPCR system, combining water-oil emulsion droplet technology with microfluidics (BioRad, 186-3005), was used and all reactions were prepared using the ddPCR Supermix for Probes (BioRad, 186-3024).

Each reaction contained a range within 0.4 ng and 848 ng of the input cfDNA as template, 450 nmol/L of each primer, and 250 nmol/L of each probe. DdPCR reaction mixes were assembled in the cells of a single-use injection molded cartridge, as follows: 8 μL of template DNA, 1.1 μL of 20X target primer/probe assay (FAM), 1.1 μL of 20X WT primer/probe assay (HEX), 11 μL of 2X ddPCR SuperMix and 0.8 μL of DNAase/RNAase-free water up to a total volume of 22 μL. Droplet generation oil (70 μL) was then loaded and the cartridge was placed into the Droplet Generator DG8 Cartridge (Bio-Rad, cat no. 186-3008). The sample and oil were then mixed under vacuum, generating mono-dispersed droplets. Forty microliters of the resulting droplet emulsion (8,000 to 16,000 droplets) were transferred by multichannel p100 pipette to an Eppendorf Twintec semi skirted 96-well PCR plate, which was then heat-sealed with pierceable foil in the PX1™ PCR Plate Sealer (BioRad) and placed in a Master Cycler thermo-cycler (Eppendorf). The cycling conditions were: 95°C for 10 minute, 40x (95° for 15 seconds, 58°C for 1 minute), then 98°C for 10 minutes (ramp rate set to 2°C/second).

The end-point fluorescence of each thermally cycled droplet was read in the QX100 droplet reader and analyzed using the Quantasoft software version 1.7 (Bio-Rad). For the quantification of the minor allele fractional abundance, the embedded “Rare Event Detection” calculation was used, which takes into account the underlying Poisson distribution to calculate the concentration of the template molecule of either allele. These values were then used to express the minor allele as a percentage of the total concentration.

### Evaluation of assay specificity and sensitivity

The specificity of each assay was evaluated as follows: (true/false) positives and (true/false) negatives were defined according to results obtained using external controls supplied by Horizon (HD780, Horizon Diagnostics, Cambridge, UK). The sensitivity was obtained using external controls supplied by Horizon (Horizon Diagnostics, HD780) harboring known EGFR mutations. The cfDNA products proposed by Horizon are all derived from human cell lines, and are fragmented to an average size of 160 bp to closely match plasma cfDNA. Furthermore, the Horizon Multiplex I cfDNA Reference Standard Set covers multiple engineered single nucleotide variants (SNVs/SNPs) with eight known mutations at 5%, 1%, and 0.1% allelic frequencies. Eight μL of Horizon's cfDNA standard were analyzed at least three times. We determined the rate of false-positivity using WT commercially available genomic DNA (Applied Biosystem, PN4344790F, Foster City, CA, USA), by analyzing the number of mutated copies in different concentrations of WT genomic DNA (from 1,000 to 2,0000 copies of WT DNA).

### Statistical analyses

All statistical calculations were done using the GraphPad InStat software (La Jolla, CA, USA). Normally distributed data were analyzed using the Student T-test or one-way ANOVA as required. Correlation between continuous variables was assessed using the Pearson test. A 2-sided P-value of < 0.05 was considered statistically significant.

## Abbreviations

cfDNA: circulating-free DNA
ctDNA: circulating tumor DNA
ddPCR: digital droplet polymerase chain reaction
EGFR: epidermal growth factor receptor
FFPE: formalin-fixed paraffin embedded
LT: Applied Biosystems, Life Technologies
NGS: next_generation sequencing
NSCLC: non-small cell lung cancer
PFS: progression-free survival
RT: room temperature
SBS: sequencing by synthesis
SAV: sequencing analysis viewer
TKI: tyrosine kinase inhibitors
WT: wild-type
